# (*E*)-3-Methyl-*N*′-(4-nitro­benzyl­idene)benzohydrazide methanol monosolvate

**DOI:** 10.1107/S1600536812003868

**Published:** 2012-02-04

**Authors:** Chun-Bao Tang

**Affiliations:** a Department of Chemistry, Jiaying University, Meizhou 514015, People’s Republic of China

## Abstract

The title hydrazone compound, C_15_H_13_N_3_O_3_·CH_3_OH, crystallized as a methanol solvate. The hydrazone mol­ecule has an *E* configuration about the C=N bond and is almost planar, with a dihedral angle between the benzene rings of 5.3 (3)°. In the crystal, the hydrazone mol­ecules are linked *via* the methanol solvent mol­ecule through N—H⋯O and O—H⋯O hydrogen bonds, so forming chains propagating along the *a*-axis direction.

## Related literature

For general background to hydrazones, see: Rasras *et al.* (2010 ▶); Pyta *et al.* (2010 ▶); Angelusiu *et al.* (2010 ▶). For related structures, see: Fun *et al.* (2008 ▶); Singh & Singh (2010 ▶); Ahmad *et al.* (2010 ▶); Tang (2010 ▶, 2011 ▶). For reference bond-length data, see: Allen *et al.* (1987 ▶).
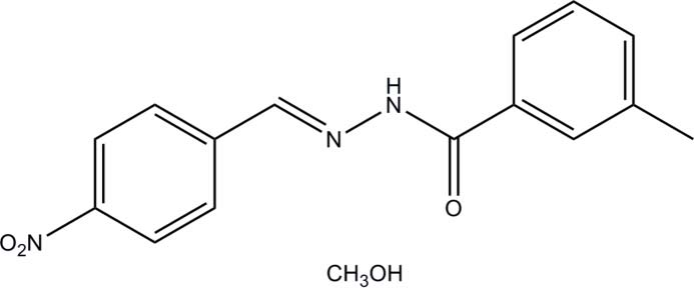


## Experimental

### 

#### Crystal data

C_15_H_13_N_3_O_3_·CH_4_O
*M* *_r_* = 315.33
Triclinic, 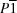

*a* = 6.581 (2) Å
*b* = 10.778 (3) Å
*c* = 11.778 (3) Å
α = 77.945 (2)°
β = 87.524 (2)°
γ = 76.146 (2)°
*V* = 793.2 (4) Å^3^
*Z* = 2
Mo *K*α radiation
μ = 0.10 mm^−1^
*T* = 298 K
0.13 × 0.10 × 0.10 mm

#### Data collection

Bruker SMART CCD area-detector diffractometer
Absorption correction: multi-scan (*SADABS*; Sheldrick, 1996 ▶) *T*_min_ = 0.988, *T*_max_ = 0.990
6084 measured reflections
3197 independent reflections
1656 reflections with *I* > 2σ(*I*)
*R*_int_ = 0.032

#### Refinement

*R*[*F* ^2^ > 2σ(*F* ^2^)] = 0.058
*wR*(*F* ^2^) = 0.142
*S* = 1.01
3197 reflections
214 parameters
1 restraint
H atoms treated by a mixture of independent and constrained refinement
Δρ_max_ = 0.16 e Å^−3^
Δρ_min_ = −0.16 e Å^−3^

### 

Data collection: *SMART* (Bruker, 2002 ▶); cell refinement: *SAINT* (Bruker, 2002 ▶); data reduction: *SAINT*; program(s) used to solve structure: *SHELXS97* (Sheldrick, 2008 ▶); program(s) used to refine structure: *SHELXL97* (Sheldrick, 2008 ▶); molecular graphics: *SHELXTL* (Sheldrick, 2008 ▶); software used to prepare material for publication: *SHELXL97*.

## Supplementary Material

Crystal structure: contains datablock(s) global, I. DOI: 10.1107/S1600536812003868/su2372sup1.cif

Structure factors: contains datablock(s) I. DOI: 10.1107/S1600536812003868/su2372Isup2.hkl

Supplementary material file. DOI: 10.1107/S1600536812003868/su2372Isup3.cml

Additional supplementary materials:  crystallographic information; 3D view; checkCIF report

## Figures and Tables

**Table 1:** Hydrogen-bond geometry (Å, °)

*D*—H⋯*A*	*D*—H	H⋯*A*	*D*⋯*A*	*D*—H⋯*A*
O4—H4⋯O3	0.82	1.98	2.767 (3)	161
N3—H3⋯O4^i^	0.90 (1)	1.98 (2)	2.869 (3)	167 (3)

Symmetry code: (i) 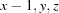.

**Fig. 1.**
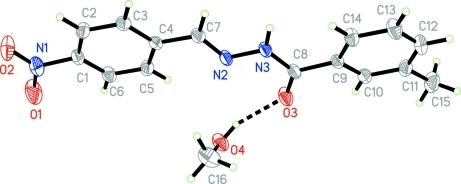
The molecular structure of the title compound, showing the atom-numbering scheme and displacement ellipsoids drawn at the 30% probability level. Hydrogen bonds are shown as dashed lines.

**Fig. 2.**
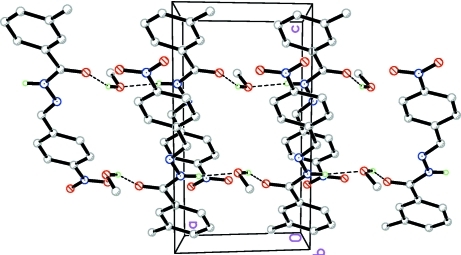
Crystal packing of the title compound, viewed along the b axis. Hydrogen bonds are shown as dashed lines.

## supplementary crystallographic information

### Comment

Hydrazone compounds have received much attention in biological and structural chemistry in the last few years (Rasras *et al.*, 2010; Pyta *et al.*, 2010; Angelusiu *et al.*, 2010; Fun *et al.*, 2008; Singh & Singh, 2010; Ahmad *et al.*, 2010). As a continuation of our work on the structural study of such compounds (Tang, 2010, 2011), the author reports herein the crystal structure of the new title hydrazone compound.

The title hydrazone molecule crystallized as a methanol solvate (Fig. 1). The methanol molecule is linked to the hydrazone molecule through an intermolecular O4—H4···O3 hydrogen bond (Fig. 1, Table 1). In the hydrazone molecule the dihedral angle between the two benzene rings is 5.3 (3)°. Bond lengths in the compound are normal (Allen *et al.*, 1987) and comparable to those in the similar compounds referred to above.

In the crystal, the hydrazone and methanol molecules are linked via the methanol molecule, through intermolecular N—H···O and O—H···O hydrogen bonds (Table 1), forming chains along the *a* axis (Fig. 2).

### Experimental

4-Nitrobenzaldehyde (0.1 mmol, 15.1 mg) and 3-methylbenzohydrazide (0.1 mmol, 15.0 mg) were dissolved in methanol (20 ml). The mixture was stirred at reflux for 10 min to give a clear yellow solution. Yellow needle-shaped crystals of the compound were formed by slow evaporation of the solvent over several days.

### Refinement

The amino H atom was located in a difference Fourier map and was refined with the N—H distance restrained to 0.90 (1) Å. Other H atoms were constrained to ideal geometries and refined as riding atoms: O—H = 0.82 Å, Csp^2^—H = 0.93 Å, and C(methyl)—H = 0.96 Å, with U_iso_(H) = k × U_eq_(parent C or O-atom), where k = 1.5 for OH and CH_3_ H-atoms and k = 1.2 for all other H-atoms.

### Crystal data

**Table:**

C_15_H_13_N_3_O_3_·CH_4_O	*Z* = 2
*M**_r_* = 315.33	*F*(000) = 332
Triclinic, *P*1	*D*_x_ = 1.320 Mg m^−^^3^
Hall symbol: -P 1	Mo *K*α radiation, λ = 0.71073 Å
*a* = 6.581 (2) Å	Cell parameters from 1091 reflections
*b* = 10.778 (3) Å	θ = 2.4–24.4°
*c* = 11.778 (3) Å	µ = 0.10 mm^−^^1^
α = 77.945 (2)°	*T* = 298 K
β = 87.524 (2)°	Cut from needle, yellow
γ = 76.146 (2)°	0.13 × 0.10 × 0.10 mm
*V* = 793.2 (4) Å^3^	

### Data collection

**Table:**

Bruker SMART CCD area-detector diffractometer	3197 independent reflections
Radiation source: fine-focus sealed tube	1656 reflections with *I* > 2σ(*I*)
Graphite monochromator	*R*_int_ = 0.032
ω scans	θ_max_ = 26.5°, θ_min_ = 2.4°
Absorption correction: multi-scan (*SADABS*; Sheldrick, 1996)	*h* = −8→8
*T*_min_ = 0.988, *T*_max_ = 0.990	*k* = −13→13
6084 measured reflections	*l* = −14→14

### Refinement

**Table:**

Refinement on *F*^2^	Primary atom site location: structure-invariant direct methods
Least-squares matrix: full	Secondary atom site location: difference Fourier map
*R*[*F*^2^ > 2σ(*F*^2^)] = 0.058	Hydrogen site location: inferred from neighbouring sites
*wR*(*F*^2^) = 0.142	H atoms treated by a mixture of independent and constrained refinement
*S* = 1.01	*w* = 1/[σ^2^(*F*_o_^2^) + (0.0521*P*)^2^ + 0.1129*P*] where *P* = (*F*_o_^2^ + 2*F*_c_^2^)/3
3197 reflections	(Δ/σ)_max_ < 0.001
214 parameters	Δρ_max_ = 0.16 e Å^−^^3^
1 restraint	Δρ_min_ = −0.16 e Å^−^^3^

### Special details

**Table:**

Geometry. All esds (except the esd in the dihedral angle between two l.s. planes) are estimated using the full covariance matrix. The cell esds are taken into account individually in the estimation of esds in distances, angles and torsion angles; correlations between esds in cell parameters are only used when they are defined by crystal symmetry. An approximate (isotropic) treatment of cell esds is used for estimating esds involving l.s. planes.
Refinement. Refinement of F^2^ against ALL reflections. The weighted R-factor wR and goodness of fit S are based on F^2^, conventional R-factors R are based on F, with F set to zero for negative F^2^. The threshold expression of F^2^ > 2sigma(F^2^) is used only for calculating R-factors(gt) etc. and is not relevant to the choice of reflections for refinement. R-factors based on F^2^ are statistically about twice as large as those based on F, and R- factors based on ALL data will be even larger.

### Fractional atomic coordinates and isotropic or equivalent isotropic displacement parameters (Å^2^)

**Table:**

	*x*	*y*	*z*	*U*_iso_*/*U*_eq_	
N1	0.1889 (4)	0.9671 (2)	0.72645 (19)	0.0595 (6)	
N2	0.0635 (3)	0.60858 (18)	0.37519 (17)	0.0428 (5)	
N3	−0.0125 (3)	0.54614 (19)	0.30155 (17)	0.0429 (5)	
O1	0.3766 (4)	0.9541 (2)	0.7382 (2)	0.0971 (8)	
O2	0.0567 (4)	1.0440 (2)	0.76810 (18)	0.0821 (7)	
O3	0.3165 (3)	0.44974 (17)	0.25767 (16)	0.0646 (6)	
O4	0.5393 (3)	0.61678 (18)	0.31310 (17)	0.0612 (5)	
H4	0.4607	0.5684	0.3120	0.092*	
C1	0.1211 (4)	0.8871 (2)	0.65514 (19)	0.0410 (6)	
C2	−0.0872 (4)	0.9129 (2)	0.6271 (2)	0.0458 (6)	
H2	−0.1851	0.9761	0.6562	0.055*	
C3	−0.1478 (4)	0.8423 (2)	0.55447 (19)	0.0431 (6)	
H3A	−0.2884	0.8582	0.5347	0.052*	
C4	−0.0020 (4)	0.7481 (2)	0.51068 (19)	0.0377 (6)	
C5	0.2076 (4)	0.7219 (2)	0.5439 (2)	0.0468 (6)	
H5	0.3062	0.6574	0.5169	0.056*	
C6	0.2688 (4)	0.7912 (2)	0.6166 (2)	0.0472 (7)	
H6	0.4081	0.7734	0.6394	0.057*	
C7	−0.0702 (4)	0.6778 (2)	0.4316 (2)	0.0436 (6)	
H7	−0.2122	0.6839	0.4225	0.052*	
C8	0.1274 (4)	0.4670 (2)	0.2444 (2)	0.0417 (6)	
C9	0.0399 (4)	0.3987 (2)	0.16696 (19)	0.0377 (6)	
C10	0.1775 (4)	0.2953 (2)	0.13095 (19)	0.0445 (6)	
H10	0.3169	0.2738	0.1544	0.053*	
C11	0.1127 (4)	0.2227 (2)	0.0608 (2)	0.0493 (7)	
C12	−0.0940 (5)	0.2591 (3)	0.0254 (2)	0.0576 (8)	
H12	−0.1409	0.2130	−0.0225	0.069*	
C13	−0.2320 (4)	0.3619 (3)	0.0593 (2)	0.0595 (8)	
H13	−0.3706	0.3844	0.0343	0.071*	
C14	−0.1669 (4)	0.4322 (2)	0.1303 (2)	0.0497 (7)	
H14	−0.2612	0.5015	0.1533	0.060*	
C15	0.2620 (5)	0.1074 (3)	0.0270 (3)	0.0771 (9)	
H15A	0.4032	0.1136	0.0362	0.116*	
H15B	0.2378	0.1065	−0.0526	0.116*	
H15C	0.2404	0.0284	0.0757	0.116*	
C16	0.4510 (5)	0.7410 (3)	0.2475 (3)	0.0846 (10)	
H16A	0.3187	0.7760	0.2802	0.127*	
H16B	0.4303	0.7347	0.1689	0.127*	
H16C	0.5433	0.7976	0.2485	0.127*	
H3	−0.1529 (16)	0.558 (3)	0.299 (2)	0.080*	

### Atomic displacement parameters (Å^2^)

**Table:**

	*U*^11^	*U*^22^	*U*^33^	*U*^12^	*U*^13^	*U*^23^
N1	0.0752 (19)	0.0521 (15)	0.0576 (15)	−0.0199 (14)	−0.0133 (14)	−0.0169 (12)
N2	0.0393 (12)	0.0414 (12)	0.0558 (13)	−0.0153 (10)	−0.0026 (10)	−0.0204 (10)
N3	0.0328 (12)	0.0458 (12)	0.0584 (13)	−0.0132 (10)	−0.0007 (11)	−0.0247 (11)
O1	0.0775 (17)	0.1043 (18)	0.133 (2)	−0.0269 (14)	−0.0248 (15)	−0.0648 (15)
O2	0.0985 (17)	0.0707 (14)	0.0876 (15)	−0.0095 (13)	−0.0094 (13)	−0.0494 (12)
O3	0.0361 (11)	0.0697 (13)	0.1027 (15)	−0.0120 (9)	−0.0018 (10)	−0.0507 (11)
O4	0.0395 (11)	0.0584 (12)	0.0944 (14)	−0.0167 (9)	−0.0036 (10)	−0.0286 (11)
C1	0.0533 (17)	0.0366 (14)	0.0373 (13)	−0.0173 (13)	−0.0053 (12)	−0.0083 (11)
C2	0.0494 (17)	0.0418 (15)	0.0481 (15)	−0.0072 (13)	0.0028 (12)	−0.0180 (12)
C3	0.0327 (14)	0.0477 (15)	0.0517 (15)	−0.0111 (12)	0.0004 (12)	−0.0146 (13)
C4	0.0355 (15)	0.0381 (13)	0.0429 (14)	−0.0137 (11)	0.0012 (11)	−0.0107 (11)
C5	0.0433 (16)	0.0456 (15)	0.0554 (16)	−0.0094 (12)	0.0018 (13)	−0.0208 (13)
C6	0.0413 (16)	0.0500 (16)	0.0550 (16)	−0.0137 (13)	−0.0076 (12)	−0.0159 (13)
C7	0.0349 (15)	0.0472 (15)	0.0540 (16)	−0.0126 (12)	−0.0011 (12)	−0.0181 (13)
C8	0.0373 (16)	0.0373 (14)	0.0555 (15)	−0.0118 (12)	−0.0010 (12)	−0.0170 (12)
C9	0.0392 (14)	0.0337 (13)	0.0437 (14)	−0.0128 (11)	0.0011 (11)	−0.0107 (11)
C10	0.0421 (16)	0.0465 (15)	0.0494 (15)	−0.0142 (13)	0.0017 (12)	−0.0157 (13)
C11	0.0600 (19)	0.0445 (15)	0.0466 (15)	−0.0124 (14)	0.0022 (13)	−0.0172 (12)
C12	0.070 (2)	0.0539 (17)	0.0579 (17)	−0.0192 (15)	−0.0130 (15)	−0.0235 (14)
C13	0.0524 (18)	0.0615 (18)	0.0689 (18)	−0.0106 (15)	−0.0204 (14)	−0.0220 (15)
C14	0.0467 (17)	0.0447 (15)	0.0605 (17)	−0.0062 (12)	−0.0069 (13)	−0.0209 (13)
C15	0.083 (2)	0.072 (2)	0.085 (2)	−0.0108 (18)	0.0072 (18)	−0.0461 (18)
C16	0.073 (2)	0.080 (2)	0.101 (3)	−0.0284 (19)	−0.0114 (19)	−0.005 (2)

### Geometric parameters (Å, º)

**Table:**

N1—O2	1.214 (3)	C6—H6	0.9300
N1—O1	1.220 (3)	C7—H7	0.9300
N1—C1	1.471 (3)	C8—C9	1.495 (3)
N2—C7	1.271 (3)	C9—C14	1.385 (3)
N2—N3	1.378 (2)	C9—C10	1.387 (3)
N3—C8	1.356 (3)	C10—C11	1.391 (3)
N3—H3	0.902 (10)	C10—H10	0.9300
O3—C8	1.225 (3)	C11—C12	1.380 (3)
O4—C16	1.401 (3)	C11—C15	1.502 (3)
O4—H4	0.8200	C12—C13	1.372 (3)
C1—C2	1.373 (3)	C12—H12	0.9300
C1—C6	1.377 (3)	C13—C14	1.381 (3)
C2—C3	1.385 (3)	C13—H13	0.9300
C2—H2	0.9300	C14—H14	0.9300
C3—C4	1.388 (3)	C15—H15A	0.9600
C3—H3A	0.9300	C15—H15B	0.9600
C4—C5	1.396 (3)	C15—H15C	0.9600
C4—C7	1.462 (3)	C16—H16A	0.9600
C5—C6	1.376 (3)	C16—H16B	0.9600
C5—H5	0.9300	C16—H16C	0.9600
			
O2—N1—O1	123.5 (2)	N3—C8—C9	116.8 (2)
O2—N1—C1	118.8 (2)	C14—C9—C10	119.1 (2)
O1—N1—C1	117.7 (2)	C14—C9—C8	124.2 (2)
C7—N2—N3	117.05 (19)	C10—C9—C8	116.8 (2)
C8—N3—N2	118.13 (19)	C9—C10—C11	121.8 (2)
C8—N3—H3	125.7 (17)	C9—C10—H10	119.1
N2—N3—H3	116.1 (17)	C11—C10—H10	119.1
C16—O4—H4	109.5	C12—C11—C10	117.6 (2)
C2—C1—C6	122.2 (2)	C12—C11—C15	121.4 (2)
C2—C1—N1	118.7 (2)	C10—C11—C15	121.0 (2)
C6—C1—N1	119.1 (2)	C13—C12—C11	121.4 (2)
C1—C2—C3	118.3 (2)	C13—C12—H12	119.3
C1—C2—H2	120.9	C11—C12—H12	119.3
C3—C2—H2	120.9	C12—C13—C14	120.6 (2)
C2—C3—C4	121.0 (2)	C12—C13—H13	119.7
C2—C3—H3A	119.5	C14—C13—H13	119.7
C4—C3—H3A	119.5	C13—C14—C9	119.5 (2)
C3—C4—C5	119.0 (2)	C13—C14—H14	120.2
C3—C4—C7	119.7 (2)	C9—C14—H14	120.2
C5—C4—C7	121.3 (2)	C11—C15—H15A	109.5
C6—C5—C4	120.3 (2)	C11—C15—H15B	109.5
C6—C5—H5	119.9	H15A—C15—H15B	109.5
C4—C5—H5	119.9	C11—C15—H15C	109.5
C5—C6—C1	119.1 (2)	H15A—C15—H15C	109.5
C5—C6—H6	120.4	H15B—C15—H15C	109.5
C1—C6—H6	120.4	O4—C16—H16A	109.5
N2—C7—C4	120.3 (2)	O4—C16—H16B	109.5
N2—C7—H7	119.8	H16A—C16—H16B	109.5
C4—C7—H7	119.8	O4—C16—H16C	109.5
O3—C8—N3	121.6 (2)	H16A—C16—H16C	109.5
O3—C8—C9	121.5 (2)	H16B—C16—H16C	109.5

### Hydrogen-bond geometry (Å, º)

**Table:**

*D*—H···*A*	*D*—H	H···*A*	*D*···*A*	*D*—H···*A*
O4—H4···O3	0.82	1.98	2.767 (3)	161
N3—H3···O4^i^	0.90 (1)	1.98 (2)	2.869 (3)	167 (3)

Symmetry code: (i) *x*−1, *y*, *z*.
